# Dr. Floyd Benton Parke

**Published:** 1914-11

**Authors:** 


Dr. Floyd Benton Parke, Buffalo, 1879, died quite suddenly September 9, at his home in Elmira. He had just finished his office work and lay down to rest before making some calls outside the house. He was born at Erin, January 27, 1856, the only son of Alexander H. Parke. After graduating from the Elmira Free Academy, he studied under Dr. O. A. Jackway of Breesport, as preceptor, in addition to attendance at the University of Buffalo. He practiced for 5 years at N. Chemung, 2 years at Erin and then came to Elmira in September, 1885. In July, 1889, he became associated with Drs. Maroney and Bush in the Elmira Drug Co. In 1883, he was elected coroner and served three years. From 1886 to 1890, he was pension examiner. He was surgeon, with rank of first lieutenant to the former 26th separate company of the National Guard and to the Elmira Fire Dept., for several years. In 1889, he became a member of the Elmira Health Dept. and was Health Commissioner from 1902 till his death. Appropriate resolutions were adopted by a joint meeting of the Elmira Academy of Medicine and the Chemung Co. Medical Society and representatives of the two societies attended the funeral in a body. Dr. Wm. S. Cain has been appointed Health Commissioner pro tempore.

